# Unlocking Cardiac Insights: Displacement of Aortic Root for Calculation of Ejection Fraction in Emergency Department in India

**DOI:** 10.5811/westjem.19394

**Published:** 2025-02-25

**Authors:** Sudhi Manu, Gopinathan Vivek, Asanaru Kunju Sanjan, A. Ajay, S. Nisarg, Mymbilly Balakrishnan Jayaraj, T. R. Aishwarya, Mohammad Khalid, S. Chetana

**Affiliations:** * Department of Emergency Medicine, Kasturba Medical College, Manipal, Manipal Academy of Higher Education, Manipal, Karnataka, India-576 104; † Department of Emergency Medicine, Kasturba Medical College Mangalore, Manipal Academy of Higher Education, Karnataka, Manipal, 576 104, India; ‡ Department of Trauma and Emergency, All India Institute of Medical Sciences, Nagpur, India; § Department of Hospital Administration, Kasturba Medical College, Manipal, Manipal Academy of Higher Education, Manipal, Karnataka, India-576 104

## Abstract

**Introduction:**

Assessing cardiac function is crucial for managing acute dyspnea. In this study we aimed to evaluate displacement of the aortic root (DAR) as a method for calculating ejection fraction (EF) in patients with undifferentiated dyspnea presenting to the emergency department (ED). The primary objective was to compare EF values obtained through DAR with the modified Simpson method, which is considered the criterion reference, within an Indian academic ED.

**Methods:**

We conducted a prospective, cross-sectional study spanning two years (December 2019–December 2021). The study enrolled 110 consecutive ED patients ≥18 years of age, presenting with undifferentiated dyspnea and normal sinus rhythm. Ultrasound-trained investigators measured DAR using M-mode ultrasonography. Experienced echocardiographers, blinded to DAR, determined EF using the modified Simpson method. Statistical analyses included the Shapiro-Wilk test, McNemar test, and the receiver operating characteristic curve.

**Results:**

The mean DAR measurement was 0.781 centimeters, with an average calculated EF of 54.4%. The EF calculated using DAR did not differ significantly from EF calculated using the modified Simpson method. Comparative analysis revealed DAR’s superior sensitivity (86.21%) compared to mitral annular plane systolic excursion (48.28%) and end-point septal separation (45.45%). The DAR method exhibited high accuracy (area under the curve = 0.958) with a cut-off value 0.706 (sensitivity 88.7%, specificity 93.1%).

**Conclusion:**

Evaluating displacement of the aortic root to calculate ejection fraction in undifferentiated dyspnea demonstrated high accuracy, sensitivity, and agreement with the modified Simpson method, which is considered the criterion reference. Its simplicity and non-invasiveness makes it a valuable initial screening tool in emergency settings, with the potential to reshape cardiac assessment approaches and optimize patient care pathways in the ED.

Population Health Research Capsule What do we already know about this issue?

*While the modified Simpson method is the criterion reference to calculate ejection fraction, simpler and more rapid tools are crucial for assessing left ventricular (LV) function in emergencies.*

What was the research question?

*Can displacement of the aortic root (DAR) accurately estimate LV ejection fraction in the ED?*

What was the major finding of the study?

*The DAR cutoff of 0.706 centimeters showed high accuracy (AUC 0.958, P < 0.001), with 88.7% sensitivity and 93.1% specificity.*

How does this improve population health?

*The DAR method offers a rapid, non-invasive EF screening tool, enhancing timely diagnosis and improving care for patients with LV dysfunction.*

## INTRODUCTION

### Background

Assessing cardiac function, particularly ejection fraction (EF), is crucial for managing acute dyspnea.^1^ ^–^ ^3^ Echocardiography is the current standard for calculating EF, but displacement of the aortic root (DAR) has emerged as a potential tool for EF calculation in patients with undifferentiated dyspnea.^2^ ^,^ ^3^ The DAR method quantifies alterations in left ventricular (LV) volume throughout the cardiac cycle, providing a surrogate measure for estimating EF.^3^ End-point septal separation (EPSS) measurement is a relatively straightforward skill that an emergency physician can acquire with minimal experience, even when confronted with regional wall motion abnormalities.^4^ ^,^ ^5^ However, measurement of LV end-systolic and end-diastolic diameters using 2D or M-mode echocardiography can pose challenges to the emergency physician in clinical practice. Tracing the endocardial border of the heart in an echocardiogram during diastole and systole is often difficult and time-consuming, especially where the wall is poorly defined.^6^ ^–^ ^10^ This approach provides clinicians with multiple options for assessing LV systolic function, catering to varying levels of expertise and clinical settings.

Mitral annular plane systolic excursion (MAPSE) assesses vertical mitral valve motion using M-mode echocardiography, measuring annular displacement towards the apex. Unlike other methods, MAPSE doesn’t require optimal endocardial definition or clear LV apex visualization, enabling broad applicability. Diminished systolic mitral valve excursion, reflected in MAPSE measurements, reliably indicates LV systolic dysfunction. The MAPSE demonstrates strong correlations, particularly in non-critically ill patients, offering effective LV function assessment even in challenging imaging scenarios.^11^ ^–^ ^15^ Emergency physicians are accurate at visual LV EF estimation without quantitative measurements, but objective measures can benefit early learners and facilitate communication.^6^ However, EF calculation using the DAR method has not been done in an Indian population in the ED setting. This highlights the need for further studies to determine DAR’s reliability and clinical applicability in the context of an Indian setting.

### Importance

Given the current limited research on the utility of DAR in Indian academic ED settings, with this investigation we aimed to fill the gap by assessing DAR’s reliability and clinical applicability. The study specifically focuses on patients with undifferentiated dyspnea, a population where EF estimation is crucial for appropriate management.

### Goal of this Investigation

Our primary objective was to calculate the EF using DAR and then compare it with EF measurements obtained through the modified Simpson method, defined as the criterion reference by the American Society of Echocardiography (ASE).^9^ ^,^ ^16^ The secondary objective was to identify the cut-off for DAR, which could predict LV dysfunction based on EF calculation. Additionally, we sought to compare the EF calculated from DAR with those obtained through EPSS and MAPSE. By evaluating DAR in comparison to the established methods, we aimed to provide insights into its potential as a reliable tool for EF estimation in the Indian setting.

## MATERIAL AND METHODS

### Study Design and Setting

This prospective, cross-sectional study was conducted across a span of two years, from December 2019–December 2021, within the ED of a teaching hospital in India. The hospital provides a broad spectrum of specialties, and its adult ED has approximately 37,200 visits annually. We obtained initial institutional research board/institutional ethics committee approval, with the registration number ECR/146/Inst/ KA/ 2013/RR-19, IEC: 1057/2019, dated May 8, 2020, and approval for study modifications on September 22, 2021. Additionally, the study is registered with the Clinical Trials Registry–India under the number CTRI/2020/10/028704, dated October 28, 2020. We adhered to ethical standards by obtaining informed consent and ensuring the voluntary participation and compliance of all subjects involved in the study. We assessed the EF of 110 patients with undifferentiated dyspnea using different methods.

### Selection of Participants and Methods of Measurements

We enrolled patients ≥18 years of age, presenting with undifferentiated dyspnea and normal sinus rhythm based on a convenience sampling. The following were excluded: patients intubated outside of a hospital; pregnant women; individuals with elevated cardiac biomarkers at presentation; those with atrial fibrillation, known valvular pathology or surgery, primary or metastatic carcinoma in the thorax; patients for whom the time between echocardiography to obtain EF using DAR and the modified Simpson method was more than 30 minutes; and those who did not provide consent. These factors could have influenced the accuracy and reliability of the EF measurements obtained through different methods. Demographic variables, including age and gender, were considered as potential confounding factors in this study.

**Figure 1.**
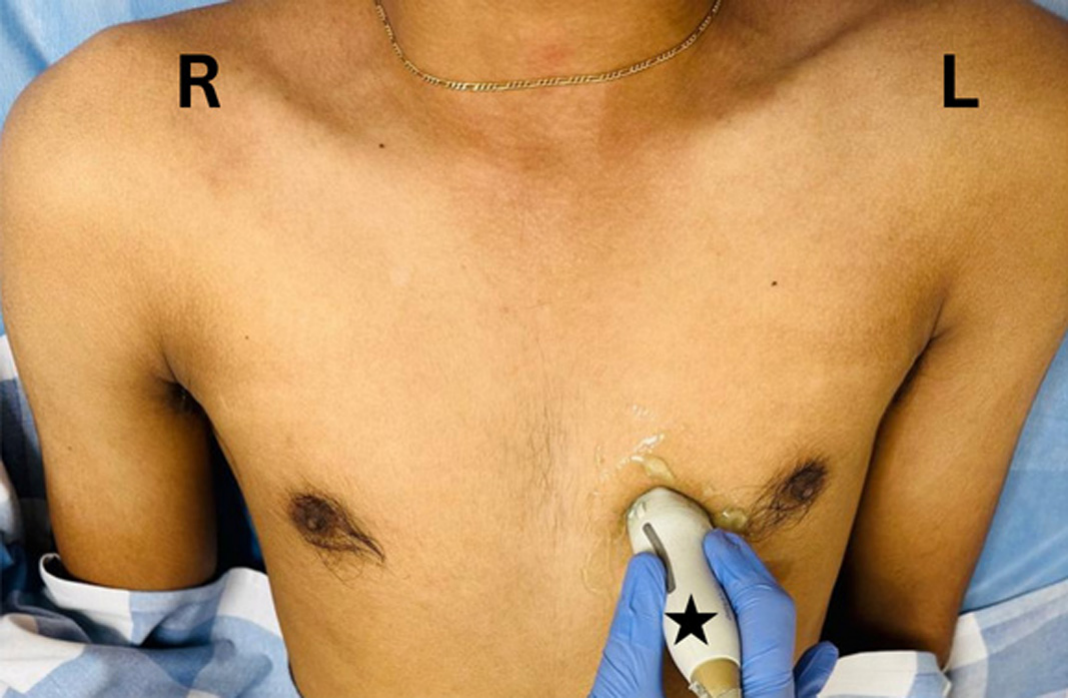
The probe is positioned in the parasternal long-axis view, with the transducer placed perpendicular to the chest wall at the third or fourth intercostal space, just to the left of the sternum, and the probe marker (black star) directed towards the patient’s right shoulder.

**Figure 2.**
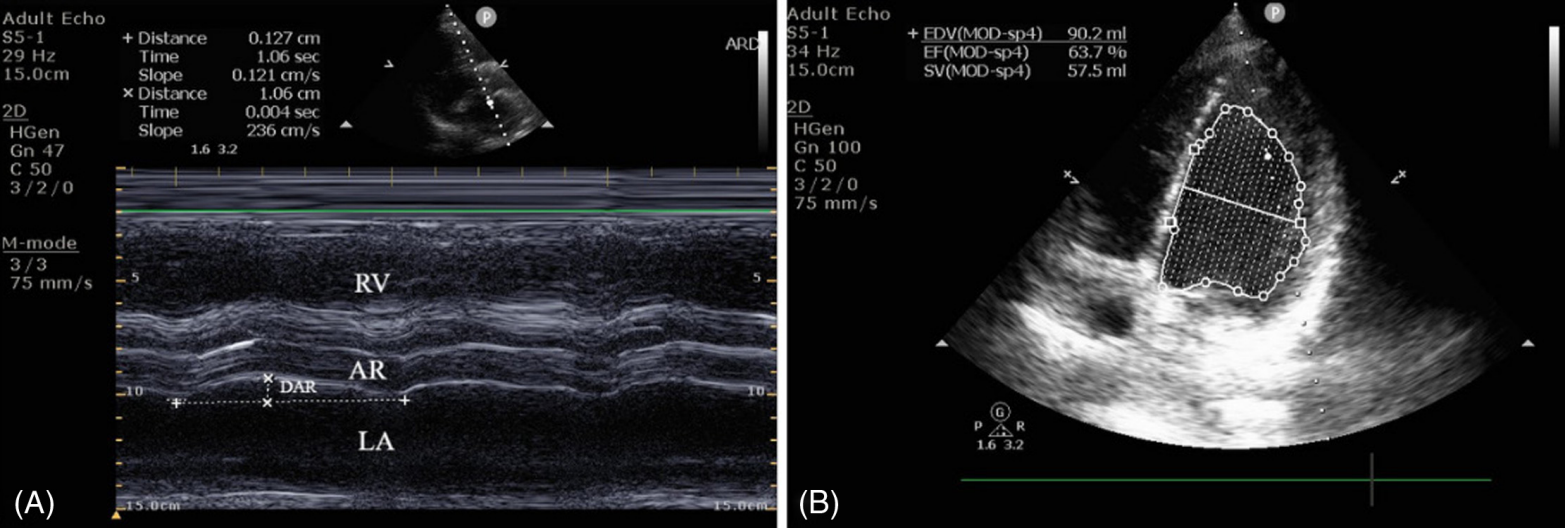
(A) We assessed ejection fraction (EF) at the bedside using M-mode ultrasonography, measuring the displacement of the aortic root (DAR) in the parasternal long-axis view. The recorded DAR for this patient was 1.06 cm. We calculated the EF using the formula (EF = 20 + 44 × 1.06), which resulted in 66.6%. (B) The echocardiographers calculated EF using the modified Simpson method [(EDV − ESV) / EDV], where [(90.2 − 32.7) / 90.2] × 100 resulted in an EF of 63.7%.

After obtaining written informed consent, the emergency clinician conducted the bedside ultrasonography proctored by the expert in point-of-care ultrasound (POCUS). Using a 3.6-megahertz micro-convex transducer, the investigator, trained in POCUS during residency training as per the curriculum, employed a Philips CX 50 ultrasound machine (Koninklijke Philips NV, Amsterdam, Netherlands) to compute the EF using DAR. Initially, 2D echocardiograms of the parasternal long-axis view were captured for DAR measurement. This view was achieved by positioning the footprint of the transducer perpendicular to the chest wall at the third or fourth intercostal space, just to the left of the sternum with the pointer towards the right shoulder (Figure 1).^17^ Optimum image required clear view of mitral valve leaflets and aortic valves. Subsequently, M-mode was placed just above the level of the aortic valve and DAR recordings were taken.^3^ The maximum anterior DAR from the horizontal axis at end-systole was measured using the leading-edge technique and recorded in centimeters (cm) (Figure 2A). The computation of EF was then done, using the formula 20 + 44 * DAR (cm).

Following the DAR measurement, the investigator calculated the EF using EPSS determined by EF = 75.5 – (2.5 × EPSS), and using MAPSE calculated by 4.8 × MAPSE (millimeters [mm]) + 5.8 for men and 4.2 × MAPSE (mm) + 20 for women.^5^ ^,^ ^14^ ^,^ ^18^ ^–^ ^21^ An experienced echocardiographer, blinded to the study procedure, evaluated LV EF using the ASE recommended Modified Simpson’s rule for this measurement (Figure 2B).^9^ ^,^ ^16^

### Outcomes

The study systematically categorized outcomes into two groups, delineating ‘normal’ EF as 50% to 70% and ‘low EF’ <50%.^22^ The primary outcome measured significant difference in calculated EF between the DAR and modified Simpsons methods. The secondary outcome of the study was to determine cut-off value of DAR with high sensitivity and specificity through receiver operating characteristic (ROC) curve analysis. Secondary outcomes also included comparison of EF calculated from DAR with that calculated from EPSS and MAPSE.

### Sample Size Calculation

With a desired margin of error of 10%, alpha error of 5%, and estimated proportion of 0.5, sample size was calculated to be 96. After considering the dropout rate of 15%, the final sample size was 110.

### Analysis

We used SPSS Statistics, version 26.0 (IBM Corp, Armonk, NY) to analyse the data. The Shapiro-Wilk test assessed normality for continuously distributed data, and we executed group comparisons in the subsequent steps. An exact McNemar test was used to identify the statistically significant changes in EF calculated using the DAR and modified Simpson’s methods. We calculated the Pearson correlation coefficient to measure strength and direction of the linear relationship between two tests. The ROC curve played a pivotal role in determining the optimal cut-off value for the validity measure of DAR.

## RESULTS

**Figure 3.**
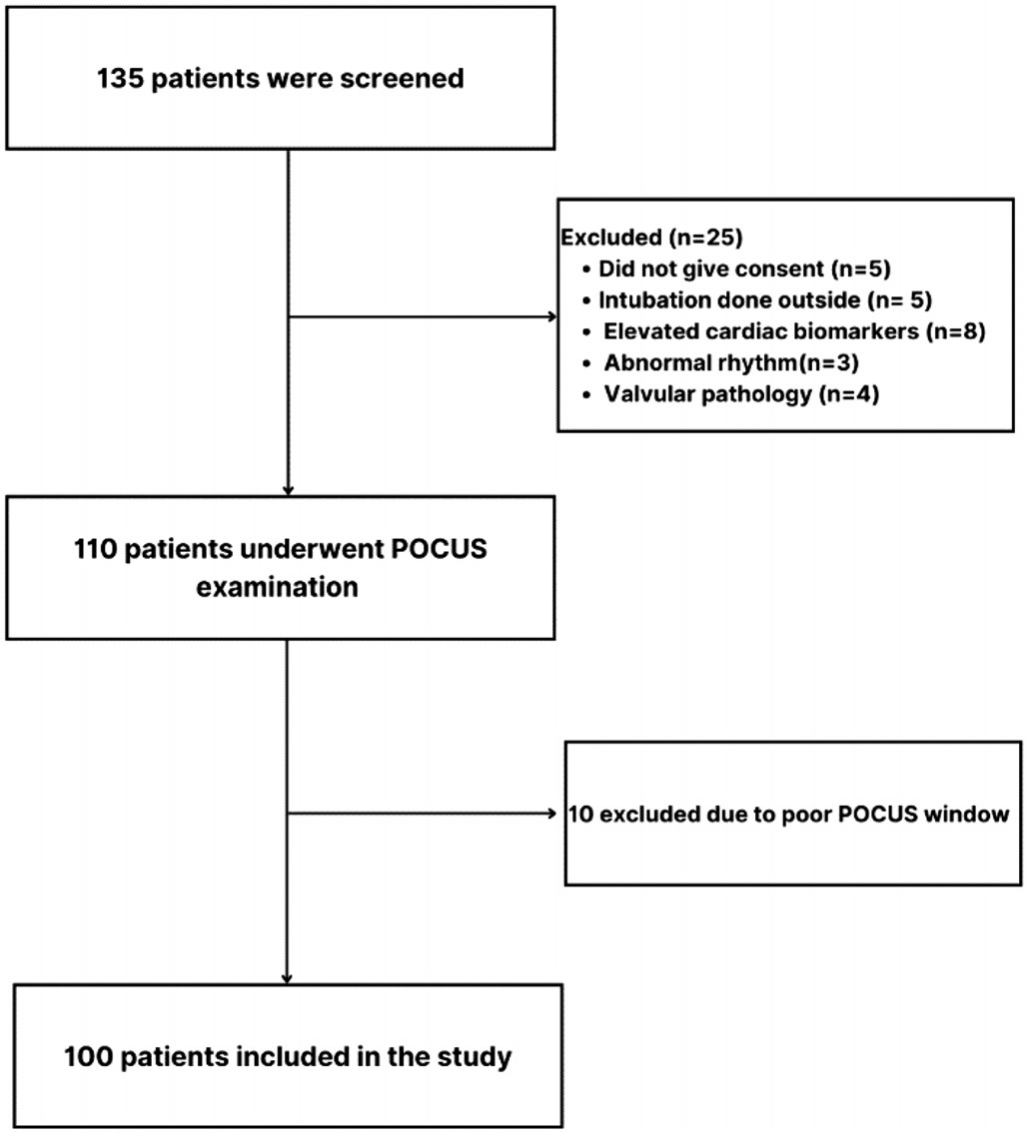
Consort patient flow diagram. *POCUS*, point-of-care ultrasound.

A total of 135 patients underwent initial screening for participation in the study. Before the POCUS assessment, we excluded 25 patients based on predefined criteria: five due to external intubation; eight with elevated cardiac biomarkers; three with abnormal rhythm; four with valvular pathology; and five who declined to participate. Following that, a POCUS examination was conducted on 110 patients, with 10 excluded due to poor image quality (Figure 3). The demographic and clinical characteristics of 100 patients who underwent POCUS, including age, heart rate, mean arterial pressure, and the mean DAR values in relation to age, gender, and comorbidities are detailed in Table 1.

**Table 1.** Demographic and clinical characteristics with displacement of aortic root mean values in 100 patients on whom point-of-care ultrasound was performed.

Patient characteristics			N = 100		
Patient age in years, mean (SD)			53.7 (16.4)		
Male, n (%)			73 (73)		
Heart rate, mean (SD)			92 (17.8)		
Respiratory rate, min-max (SE)			20–36 (0.31)		
MAP, mean (SD), mm Hg			90.6 (16.7)		
Symptoms					
Fever, n (%)			33 (33)		
Cough, n (%)			33 (33)		
Chest pain, n (%)			14 (14)		
Comorbidities					
Type II diabetes mellitus, n (%)			36 (36)		
Systemic hypertension, n (%)			46 (46)		
IHD, n (%)			16 (16)		
Cardiomyopathy, n (%)			2 (2)		
Oxygen requirement					
Nasal prongs (2L–4L), n (%)			26 (26)		
Face mask (6L–10L), n (%)			59 (59)		
Non-rebreathing mask (>10L), n (%)			14 (14)		
**Category**	**Subgroup**	**n (%), N = 100**	**Mean DAR**	**Standard deviation**	***P*-value**
Age group	< = 30 years	6 (6%)	1.04	0.05	
	31–40 years	12 (12%)	0.94	0.29	
	41–50 years	19 (19%)	0.83	0.26	
	51–60 years	18 (18%)	0.73	0.28	
	61–70 years	26 (26%)	0.68	0.29	
	71–80 years	16 (16%)	0.80	0.22	
	>80 years	3 (3%)	0.50	0.19	
Gender	Male	73 (73%)	0.78	0.29	0.76
	Female	27 (27%)	0.78	0.23	
Comorbidities	Type II diabetes mellitus (yes)	36 (36%)	0.72	0.30	0.16
	Type II diabetes mellitus (no)	63 (63%)	0.82	0.26	
	Systemic hypertension (yes)	46 (46%)	0.69	0.28	0.001
	Systemic hypertension (no)	54 (54%)	0.86	0.25	
	IHD (Yes)	15 (15%)	0.53	0.22	<0.001
	IHD (No)	85 (85%)	0.82	0.26	

*min*, minimum; *max*, maximum; *SD*, standard deviation; *SE*, standard error; *MAP*, mean arterial pressure; *IHD*, ischemic heart disease.

In this study we observed a mean DAR measurement of 0.781 cm (SD 0.277 cm) and an average calculated EF of 54.4% (SD 12.2%). The Pearson correlation coefficient was calculated to measure strength and direction of the linear relationship between two tests and was found to be 0.81, which suggests a strong positive relation between the results. The study conducted an exact McNemar test to identify statistically significant variations in abnormal and normal EF distribution between the EF calculated using DAR/MAPSE/ EPSS and the EF measured by an echocardiographer (criterion reference), as outlined in Table 2. The statistical analysis revealed a lack of significant differences (*P* = 0.39) between the EF calculated using DAR and the EF measured by echocardiography.

**Table 2.** Comparative analysis of ejection fraction (EF) measurements using DAR, MAPSE, and EPSS* against actual EF by the modified Simpson method.

		Actual EF by modified Simpson method			
Calculated EF		Abnormal, n (%) N = 100	Normal, n (%) N = 100	Total, N (%)	*P-*value
Calculated EF with DAR	Abnormal	25 (25%)	8 (8%)	33 (33%)	0.39
	Normal	4 (4%)	63 (63%)	67 (67%)	
Calculated EF with MAPSE	Abnormal	14 (14%)	3 (3%)	17 (17%)	0.01
	Normal	15 (15%)	68 (68%)	83 (83%)	
Calculated EF with EPSS	Abnormal	17 (17%)	2 (2%)	19 (19%)	0.01
	Normal	12 (12%)	69 (69%)	81 (81%)

*EF*, ejection fraction; *DAR, displacement of aortic root; *MAPSE*, mitral annular plane systolic excursion; *EPSS*, end-point septal separation.

**Figure 4.**
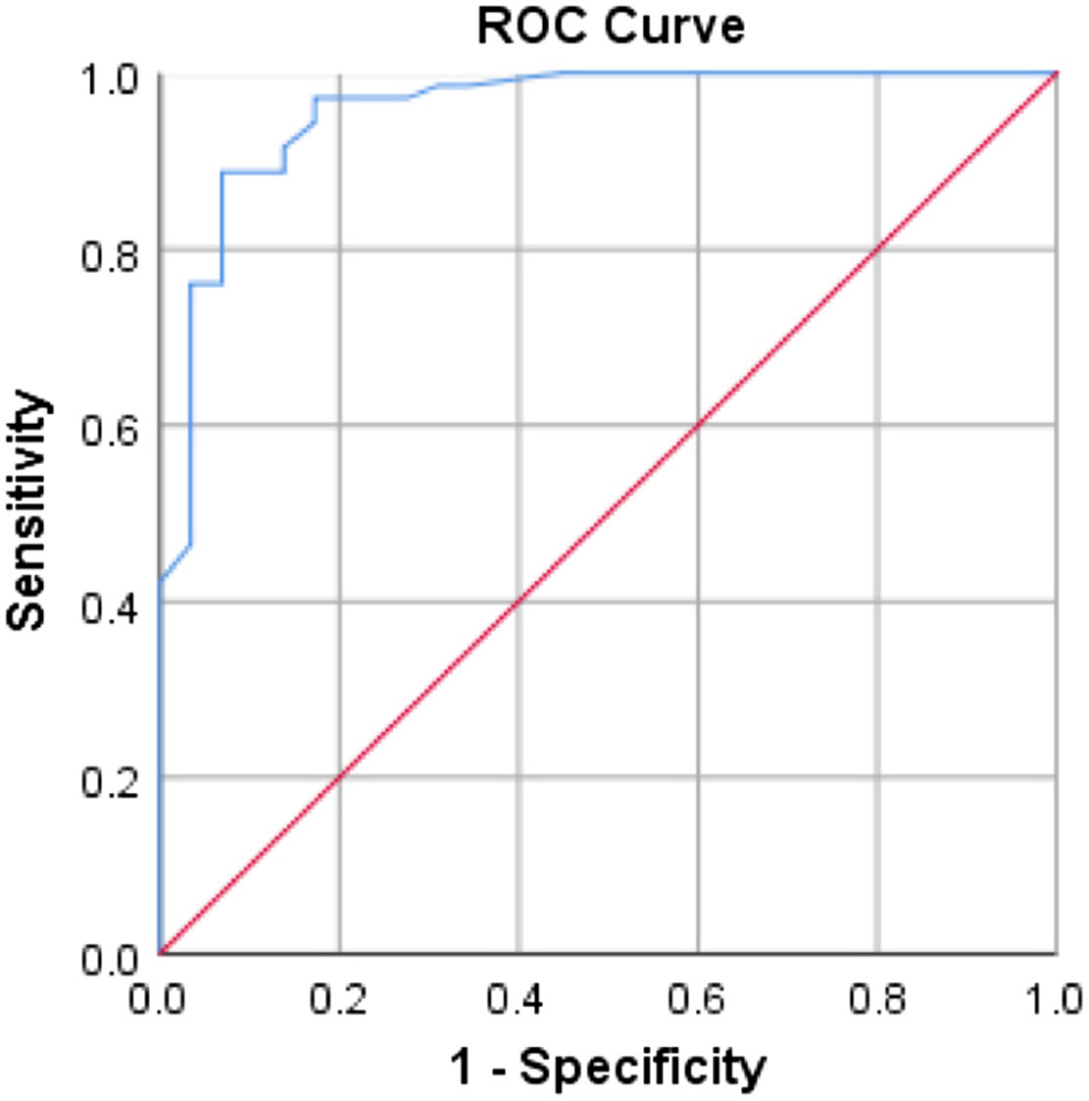
The ROC curve of the sensitivity of displacement of aortic root for ejection fraction when the cut-off value is 0.70 centimeters. Area under the ROC curve = 0.958 (95% confidence interval 0.914–1.000, P < 0.001 for predicting EF). *ROC*, receiver operating characteristic.

We conducted ROC curve analysis, which demonstrated DAR’s validity with a high accuracy reflected in an area under the curve (AUC) of 0.958 (95% confidence interval [CI] 0.914–1.000, *P* < 0.001) for predicting EF. The optimal cut-off point for DAR was identified as 0.706, providing a sensitivity of 88.7%, specificity of 93.1%, LR+ (likelihood ratio) of 12.86, and LR- of 0.12. (Figure 4). The Pearson correlation coefficient calculated for EF calculated by MAPSE and the modified Simpson method was 0.54 and that of EPSS and the modified Simpson method was 0.76. For calculated EF with MAPSE, 48.3% of patients were categorized as having abnormal EF, exhibiting a statistically significant difference compared to EF calculated by the modified Simpson method (*P* = 0.01) (Table 2). Similarly, calculated EF with EPSS demonstrated a comparable discordance, with 58.6% classified as abnormal, significantly differing from EF calculated by the modified Simpson method (*P* = 0.01) (Table 2).

Table 3 presents a comparative assessment of the efficacy of EF measurements using MAPSE, EPSS, and DAR against the criterion reference. The sensitivity of DAR is notably higher than MAPSE and EPSS, which suggests that it is a better screening tool. Calculated EF from DAR obtained highest negative predictive value (NPV), suggesting a better ability to correctly identify patients with normal EF.

**Table 3.** Comparative efficacy of ejection fraction measurements using MAPSE, EPSS, and DAR* against the criterion reference with 95% confidence intervals.

	Calculated EF with MAPSE (95% CI)	Calculated EF with EPSS (95% CI)	Calculated EF with DAR (95% CI)
Sensitivity	48.3% (39.2–57.4)	45.5% (36.2–54.8)	86.2% (79.7–91.4)
Specificity	95.8% (90.3–98.4)	97.0% (94.4–99.4)	88.7% (83.3–92.9)
PPV	82.4% (73.9–89.3)	88.2% (83–93.3)	75.8% (68.0–82.1)
NPV	81.9% (74.3–87.5)	78.3% (71.4–85.2)	94.0% (90.2–96.7)
Accuracy	82.0% (74.8–87.9)	80.0% (73.1–86.9)	88.0% ((82.2–92.8)

*EF*, ejection fraction; *MAPSE*, mitral annular plane systolic excursion, *EPSS*, end-point septal separation; *DAR, displacement of aortic root; *CI*, confidence interval; *PPV*, positive predictive value; *NPV*, negative predictive value.

## DISCUSSION

Dyspnea is a common presenting complaint in the ED, accounting for approximately 5% of all ED presentations in the Asia-Pacific region.^23^ ^,^ ^24^ Emergency physicians frequently face the challenge of making swift diagnoses and developing treatment plans based on limited clinical information.^25^ ^,^ ^26^ Point-of-care ultrasound has become a standard component of routine clinical examinations in the ED, enhancing the management of dyspnea by facilitating the diagnosis of its underlying causes.^27^ Similarly, evaluating LVEF through echocardiography plays a crucial role in diagnosing and managing a wide range of patients in the ED, further emphasizing the importance of ultrasound in emergency care.^28^ Most research in the ED has emphasized visual assessments of LVEF instead of relying on calculations derived from measuring the dimensions of the LV chamber across the cardiac cycle.^29^ ^–^ ^31^

This study addresses a crucial aspect of emergency care by exploring the assessment of LV function in patients with undifferentiated dyspnea. While the modified Simpson method remains the criterion reference, investigating the potential of DAR as an alternative method opens avenues for expedited and more accessible evaluations in time-sensitive environments like the ED. As a non-invasive and easily accessible tool, DAR has shown promise in accurately predicting LVEF, making it valuable for identifying patients at risk of LV dysfunction.^3^ ^,^ ^32^ The DAR method showed an accuracy rate of 88% in correctly classifying LV dysfunction, demonstrating its clinical applicability in emergency settings. This rate surpasses the accuracy of MAPSE and EPSS assessments for LV dysfunction, including the 75% accuracy reported in a study by Schick et al.^33^

This study’s robust methodology and compelling results substantially contribute to establishing the validity and clinical relevance of DAR. The DAR method exhibits good sensitivity (86.2%) and specificity (88.7%) and has a positive correlation with the values of EF obtained through the modified Simpson method. This sensitivity and specificity are consistent with the findings of Ünlüer et al, who reported 94.4% and 94.1%, respectively.^3^ The increased sensitivity of DAR compared to EPSS and MAPSE in our study makes it a valuable tool for the early detection of LV dysfunction in emergency settings.These findings indicate that emergency physicians can use DAR as a valuable alternate tool for assessing the LV function at the bedside.^33^ In the ED, where rapid decision-making is crucial, DAR can be incorporated as an initial screening tool to identify patients with compromised LV function, guiding further diagnostic testing, management, interventions or specialist referrals.

When comparing DAR with traditional methods, MAPSE showed a sensitivity of 48.3% (95% CI 39.2–57.4) and specificity of 95.8% (95% CI 90.3–98.4), while EPSS exhibited a sensitivity of 45.5% (95% CI 36.2–54.8) and specificity of 97.0% (95% CI 94.4–99.4). These results contrast with prior studies, such as that by McKaigney et al, who observed significantly higher EPSS sensitivity (83.3%) but much lower specificity (50.0%), and Schick et al, who reported MAPSE sensitivity of 42% and specificity of 89%.^18^ ^,^ ^33^ The higher sensitivity (83.3%) and lower specificity (50.0%) of EPSS reported by McKaigney et al may stem from their comparison of EPSS with EF calculated using the Teichholz method. Folland et al found that EF calculated through the modified Simpson method demonstrated better correlation with radionuclide ventriculography than the Teichholz method, with correlation coefficients (r values) of 0.75 and 0.46, respectively. Furthermore, the ASE no longer recommends the Teichholz method for calculating LV volumes.^9^ ^,^ ^34^ The higher specificity of MAPSE and EPSS in our study suggests that these measurements are more effective in confirming LV dysfunction than in detecting it, underscoring the utility of DAR’s higher sensitivity for early identification.

The DAR offers a practical advantage in the ED setting due to the straightforward visualization of the aortic root compared to LV structures, making it easier to measure under challenging conditions. Furthermore, the motion of the aortic root resembles the left atrial volume curve, suggesting that its movement, influenced by its attachment to the cardiac skeleton, may reflect the dynamics of left atrial filling and emptying.^35^ ^–^ ^37^ The observed correlation between DAR and stroke volume suggests that DAR measurements may calculate LVEF effectively, providing valuable insights into cardiac performance. Lower DAR values were consistently associated with conditions linked to reduced stroke volume and EF, highlighting DAR’s relevance in assessing patients with undifferentiated dyspnea and potentially compromised cardiac function.

The DAR’s high NPV enhances its reliability in excluding patients with normal EF, which is crucial for determining appropriate next steps in ED care. The EPSS exhibited the highest positive predictive value, emphasizing its role in confirming reduced EF. However, DAR’s combined sensitivity and NPV make it a more comprehensive tool for initial screening, ensuring that patients with likely normal cardiac function are appropriately triaged. Despite its advantages, DAR should not be seen as a replacement for all echocardiographic assessments but rather as a complementary tool, especially in time-limited environments. Its heightened sensitivity compared to MAPSE and EPSS, combined with its rapid application, makes it a promising option for emergency physicians. However, further research and validation are required to establish DAR’s broader applicability in diverse patient populations and settings.

## LIMITATIONS

While the results are promising, this study has limitations. It was conducted within a single-center environment, potentially limiting the generalizability of the findings. A multicenter study involving diverse patient populations would provide more robust validation. Additionally, the study doesn’t delve into the causes of dyspnea, which can vary widely and might influence the applicability of DAR in different scenarios. We excluded 9% of patients from this study due to a poor POCUS window. Patients enrolled in this study exhibited exclusively regular cardiac rhythms. Although each M-mode recording of the aortic root (AR) had the potential to encompass multiple cardiac cycles for DAR calculation, it is crucial to emphasize that the extent of AR displacement consistently remains notable across all cardiac cycles in individuals with regular heart rhythms. When patients exhibit irregular heart rhythms, a potential adaptation could involve calculating the average DAR measurement over three to five cardiac cycles. This adjustment could enhance the accuracy of measurements in such cohorts. Future research initiatives could delve deeper into investigating and addressing this particular aspect.

## CONCLUSION

DAR emerges as an efficient and reliable method for rapid EF assessment, providing emergency physicians with a valuable tool for bedside evaluation of LV function, especially when time and resources are limited. This paves the way for integrating DAR into emergency protocols and routine emergency clinical practice. While these findings are promising, we acknowledge the need for prospective validation in a diverse patient population.
